# Morphology and Chemical Purity of Water Suspension of Graphene Oxide FLAKES Aged for 14 Months in Ambient Conditions. A Preliminary Study

**DOI:** 10.3390/ma14154108

**Published:** 2021-07-23

**Authors:** Adrian Chlanda, Krystian Kowiorski, Marcin Małek, Ewa Kijeńska-Gawrońska, Monika Bil, Małgorzata Djas, Tomasz Strachowski, Wojciech Swieszkowski, Ludwika Lipińska

**Affiliations:** 1 Research Group of Graphene and Composites, Łukasiewicz Research Network-Institute of Microelectronics and Photonics, Aleja Lotników 32/46, 02-668 Warsaw, Poland; malgorzata.djas@imif.lukasiewicz.gov.pl (M.D.); tomasz.strachowski@imif.lukasiewicz.gov.pl (T.S.); ludwika.lipinska@imif.lukasiewicz.gov.pl (L.L.); 2 Research Group of Functional Materials, Łukasiewicz Research Network-Institute of Microelectronics and Photonics, Aleja Lotników 32/46, 02-668 Warsaw, Poland; krystian.kowiorski@imif.lukasiewicz.gov.pl; 3 Faculty of Civil Engineering and Geodesy, Military University of Technology, Gen. Sylwestra Kaliskiego 2, 00-908 Warsaw, Poland; marcin.malek@wat.edu.pl; 4 Centre for Advanced Materials and Technologies CEZAMAT, Warsaw University of Technology, Poleczki 19, 02-822 Warsaw, Poland; ewa.kijenska@pw.edu.pl (E.K.-G.); monika.bil@pw.edu.pl (M.B.); 5 Faculty of Materials Science and Engineering, Warsaw University of Technology, Biomaterials Group, Wołoska 141, 02-507 Warsaw, Poland; wojciech.swieszkowski@pw.edu.pl; 6 Faculty of Chemical and Process Engineering, Warsaw University of Technology, Waryńskiego 1, 00-645 Warsaw, Poland

**Keywords:** graphene oxide flakes, nanomaterial, chemical composition, microscopical characterization

## Abstract

Graphene and its derivatives have attracted scientists’ interest due to their exceptional properties, making them alluring candidates for multiple applications. However, still little is known about the properties of as-obtained graphene derivatives during long-term storage. The aim of this study was to check whether or not 14 months of storage time impacts graphene oxide flakes’ suspension purity. Complementary micro and nanoscale characterization techniques (SEM, AFM, EDS, FTIR, Raman spectroscopy, and elemental combustion analysis) were implemented for a detailed description of the topography and chemical properties of graphene oxide flakes. The final step was pH evaluation of as-obtained and aged samples. Our findings show that purified flakes sustained their purity over 14 months of storage.

## 1. Introduction

Graphene is a two-dimensional, one-atom-thick layer of carbon, in which atoms are oriented in a hexagonal crystal lattice. The promising and appealing properties of graphene-based materials are widely used in many research-oriented fields, like fuel cells [1,2], batteries [3,4], screens [5,6], sensors [7,8], flexible electronics [9,10], tissue engineering [11,12], and membranes [13,14]. Moreover, their unique features result in an increase of materials’ mechanical strength, electrical and thermal conductivity, and surface area and flexibility [15,16,17], making them the most preferred choice in experimental procedures. Despite many scientific papers highlighting the important role of graphene in shaping material properties, little is known about time-dependent changes occurring during its long-term storage.

Graphene oxide (GO), one of graphene’s derivatives, which undergoes oxidation with oxygen-containing functional groups, is gaining popularity. However, it should be addressed that it possesses not only lower electrical conductivity compared to pristine graphene, but is also more hydrophilic. Moreover, pristine GO flakes are susceptible to light and temperature fluctuations [18,19,20]. For example, Xue et al. pointed out that UV light can induce a photochemical reduction of graphene oxide into reduced graphene oxide (RGO) [21]. Detailed examination, including Raman spectroscopy, Fourier transform infrared spectroscopy, X-ray diffraction, X-ray photoelectron spectroscopy, as well as an ultraviolet and visible spectrophotometer, proved that GO can be effectively reduced by UV light (395 nm), with the intensity of 100 mW/cm^2^ even at room temperature.

As stated above, apart from light, temperature is another factor influencing GO reduction. It governs the removal of oxygen from the GO surface, and consequently, the effectiveness of the reduction of GO flakes is prompted. An abrupt increase of temperature leads to the crystallization of the graphene oxide, thus improving the resulting material’s electrical conductivity [22,23]. It is worth reminding that during reduction, oxygenated functional groups of pristine GO flakes are eliminated, and that enables to tune properties of graphene flakes. The difference between GO and RGO manifests mainly in their conductivity and morphology. It is well established that GO flakes are typically characterized by a wrinkled surface, which is related to the presence of oxygen-containing functional groups [24]. Reduced graphene flakes are not flat and consist of crumpled sheets, which are closely associated with each other. Of particular interest is the fact that together with the density of oxidizing species, GO flakes’ morphology is one of the crucial factors governing the restoration of an sp2 graphene plane from an sp3 configuration [25]. At the same time, GO is an insulator, while RGO is electrically conductive, with the conductivity depending on the degree of reduction. Conductivity alterations, in this case, may lead to different applications of the resulting material—a pristine one (GO) or reduced (RGO).

Another interesting discovery related to the scope of this study was described by Yang et al. [26], who proved that the aging of nanocarbons (fullerenes, single- and multi-walled carbon nanotubes) can influence their surface area, pore-volume, structural defects, and amount of oxygen present on the surface of such materials, and thus changing properties of nanocarbons. Interestingly, different oxygen content of GO may significantly alter the mechanical, electrical, and chemical properties of such material [27,28,29]. This state of affairs opens up new opportunities for tuning properties of resulting graphene-based materials by varying their oxidation degree. Having this in mind, we presume that studying aging-related alterations of graphene derivatives can be of great interest for practical purposes, as materials’ properties and performances are some of the most important issues regarding different types of commercially available products [30,31,32]. The primary motivation upon which this study relies is to check whether or not storage time alters the morphology and ionic conductivity of graphene oxide flakes.

The research aims to investigate long-term storage on water suspension of graphene oxide flakes’ properties. For this purpose, GO flakes dispersed in water were conditioned in a closed container for over 14 months in ambient conditions without light access. Micro- and nanoscale observations using scanning electron microscopy (SEM) and atomic force microscopy (AFM) were followed by chemical properties evaluation (Fourier transformed infra-red, Raman spectroscopy, and pH measurements) of four types of water solution of GO flakes samples, namely: as-obtained (containing after-preparations impurities inclusions), purified (samples subjected to purification after fabrication), aged_as-obtained (containing after-preparations impurities inclusions and conditioned for 14 months), and aged_purified (samples subjected to purification after fabrication and conditioned for 14 months). This approach allowed us to get a deeper insight into GO flakes alterations during 14 months of their storage, which can be useful while designing and fabricating graphene-based products for specific needs.

## 2. Materials and Methods

### 2.1. Synthesis and Purification of Graphene-Oxide Flakes

**Figure 1:**
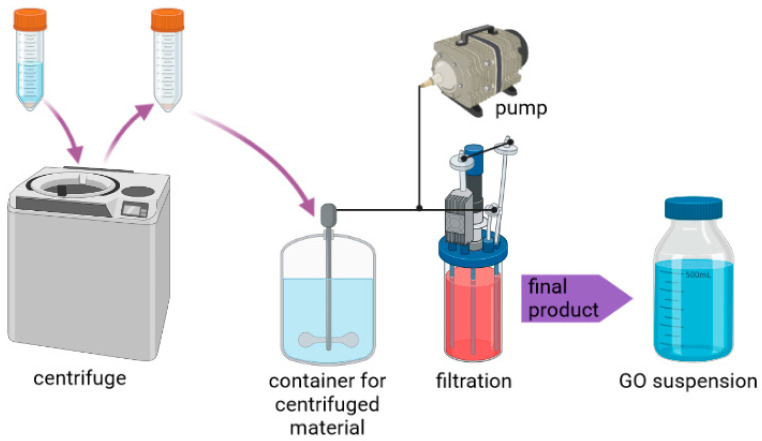
A scheme depicting purification procedure implemented in this study.

Modified Hummers method [33,34] was utilized in order to synthesize graphene oxide (GO) flakes. Sixty grams of graphite flakes (125–150 μm in diameter) purchased from Asbury Carbons (USA) were placed in a beaker filled with solution of 34 g of potassium nitrate (101.10 g/mol KnO_3_-pure, Chempur, Poland) in 4 L of sulfuric acid (95% H_2_SO_4_-pure, Chempur, Poland). After mixing, the beaker of solution was transferred into an ice bath. Subsequently, 360 g of potassium permanganate (158.04 g/mol KMnO_4_-pure, Chempur, Poland) was added into the resulting solution. Next, the beaker was removed from the ice bath and was stirred for 3 h. The temperature of the solution was controlled so as not to exceed 30–35 °C. After 3 h, the solution was left to cool down at room temperature (25 °C). The next step was the addition of the deionized water. Still, the temperature was controlled, as we did not want it to exceed 35 °C. The following stage of the synthesis of the material consisted of heating and stirring of acid–graphite oxide mixture to 95 °C for 15 min. After that time, the beaker was set to reach room temperature. The final step of the oxidation of the material was the addition of hydrogen peroxide (30% H_2_O_2_-pure, Chempur, Poland) and deionized water. Graphite was exfoliated via sonication for 1 h with ultrasonic processor (Sonics and Materials INC, VCX750, Newtown, CT, USA). The last step was the purification of the resulting material by via centrifugation (Thermo Lynx 4000, Osterode, Germany). After centrifugation, the resulting material was put in a stirred container, and filtered using cross-flow membrane filtration (24 h in a room temperature) (Figure 1).

### 2.2. Aging of Graphene-Oxide Flakes

The as-obtained and purified samples, in the form of an aqueous dispersion of GO flakes, were stored in a tightly screwed glass bottle for 14 months at ambient temperature without access to the light. All the samples synthesized and tested in this study, both fresh and aged, were stored at 4 g/L concentration.

### 2.3. Atomic Force Microscopy

MFP 3D BIO atomic force microscope, purchased from Asylum Research/Oxford Instruments was operating in Semi-Contact mode in order to visualize topography of synthesized materials and for the evaluation of flakes’ thickness. Samples for the AFM examination were prepared in the form of water suspension. Such suspension was pipetted mica surface (Ted Pella). Subsequently the samples were stored in a Memmert VO 200 vacuum dryer for 1 h at 50 mbar prior to imaging.

Surface visualization was performed in the ambient conditions (relative humidity of 18% and temperature of 22 °C). AC 160 TS R3 (Olympus) scanning probe (spring constant of c.a. 26 N/m and radius of c.a. 10 nm–according to the microscope’s producer) was mounted in the microscope. Prior to visualization, the scanning probe was calibrated with Auto Tune method in order to set its drive frequency (c.a. 320 kHz). AFM topography images were recorded at 0.6 Hz scan rate. Analysis of the recorded topographical maps were performed using IgorPro ver. 6.17.

### 2.4. Scanning Electron Microscopy (SEM) and Elemental Analysis (EDS)

The morphology of graphene oxide flakes was determined using a scanning electron microscope (SEM) Auriga CrossBeam Workstation (Carl Zeiss). Samples were prepared in the following manner: a water-diluted GO flakes solution was applied on the silicon wafers (previously treated with piranha solution—a mixture of sulfuric acid and 30% hydrogen peroxide (3:1)) and dried in a vacuum dryer at the temperature of 40 °C for up to 5 h. Samples were not sputtered prior to imaging.

In the case of elemental analysis, the water suspension of the flakes, prepared at a concentration of 0.4 mg/mL, was poured into a Teflon mold (50 mm in diameter) and left under a laminar flow chamber for 72 h. Consequently, the air-dried samples in the form of thin films were freeze-dried at the temperature of −20 °C for up to 72 h using an Alpha 1–2 (Christ) lyophilizator. The resulting specimens were then mounted onto SEM tables and evaluated using Phenom Pro X (FEI), equipped with an EDS spectrometer. To avoid any unwanted impurities, samples were not coated before the examination.

### 2.5. FTIR

Attenuated total reflectance Fourier transform spectroscopy analysis (ATR FTIR) on graphene oxide flakes was made using Nicolet 8700 FTIR (Thermo Scientific, Waltham, MA, USA) over a range of 4000–400 cm^−1^ at a resolution of 4 cm^−1^. To prepare the samples in the form of thin films for analysis, the water suspension of the flakes in the concentration of 0.4 mg/mL was poured into a Teflon mold of 50 mm diameter and left under a fume hood for 72 h. The air-dried samples were thereafter freeze-dried at −20 °C for up to 72 h using an Alpha 1–2 lyophilizator.

### 2.6. Raman Spectroscopy Analysis

Raman spectra were recorded on Renishaw in Via Raman Microscope using a 2400 L/mm grating and a 20× objective lens. Samples for Raman analysis were prepared in the same manner as for SEM. Ar green laser with 514 nm wavelength was used to excite Raman signal. An integration time of 3 s was used, and 15 accumulations were taken for each spectrum. Raw data were processed with Wire 3.54 Renishaw software. The position of D and G peaks were determined by a Gaussian/Lorentzian fit after baseline subtraction.

### 2.7. pH Evaluation

Four samples of the aqueous suspension of GO were prepared (200 mL each with a concentration of 4 g/L) in order to examine the suspensions’ pH. The pH-meter was calibrated using calibration samples of known pH delivered by the apparatus producer. The measurement was carried out by immersing the pH-meter’s probe (Elmetron, model CP-401) in the solution (temp. 25 °C). The measurement was repeated ten times.

### 2.8. Elemental Combustion Analysis

Elemental analysis was utilized along with the experimental protocol presented in our previous work [34]. For that purpose, we implemented a setup composed of three combustion analyzers: CHN628 (working temperature of 950 °C), CHN628S (working temperature of 1350 °C) and OH836 (working temperature of 950 °C)—all purchased from Leco. Prior to experiment, the analyzers were calibrated with appropriate calibration standards of known chemical composition. The CHN628 analyzer was set to detect carbon, CHN628S-sulfur and the OH836-oxygen. Three samples of each material were tested in order to acquire adequate statistical information.

### 2.9. X-ray Diffraction Analysis

X-ray diffraction XRD analysis was performed using Rigaku Diffractometer, equipped with Cu Kα anode of 8.038 keV. Examination parameters were set as follow: scanning speed: 2 deg/min, IC = C30 mA, UC = C40 kV and sampling density: 0.02 deg. The samples were prepared in the form of thin films.

### 2.10. Statistical Analysis

For the statistical analysis made with GraphPad Prism (ver. 7.04) software, measurements were triplicated. In order to acquire adequate results, we utilized one-way ANOVA with additional post-hoc Tukey’s. The differences were described as statistically significant for p values lower than: * *p* < 0.05, ** *p* < 0.01, *** *p* < 0.001, **** *p* < 0.0001. We want to inform that the data visualized in the manuscript are shown as mean values with their standard deviation.

## 3. Results and Discussion

**Figure 2:**
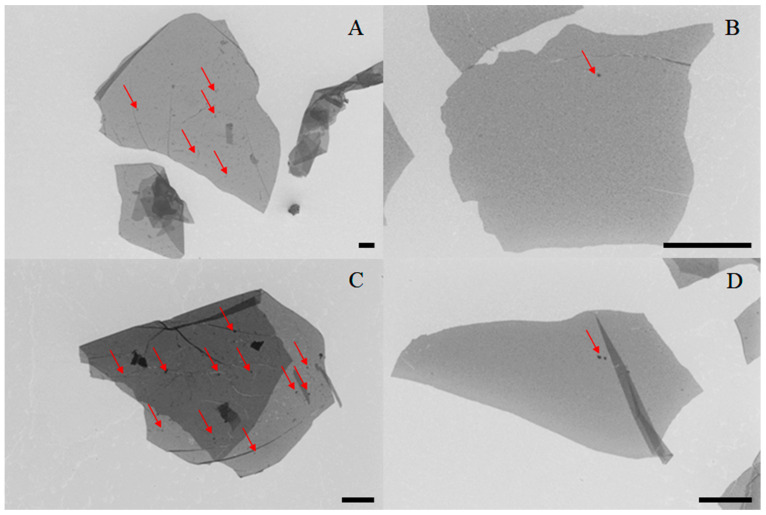
SEM images of graphene oxide flakes: (**A**) as-obtained, (**B**) purified, (**C**) aged_as-obtained, (**D**) aged_purified. Scalebar normalized to 2 µm. The red arrows indicate impurities on the flakes’ surface.

Scanning electron microscopy images permitted us to determine the degree of contamination of tested samples (Figure 2). Regardless of the flakes’ shape and size, samples deprived of purification contained more impurities on their surface than samples that underwent purification. Although such a result was expected, it is worth underlining that the purification procedure implemented herein guaranteed production of high-quality flakes (evidenced by EDS and elemental combustion analysis). After 14 months of storage more impurities were registered on the surface of the flakes deprived of purification, whereas samples that were purified and aged stayed uncontaminated. In addition, analysis of flakes’ diameter for all the materials described in the manuscript was performed and we did not observe any storage-related differences (Figure S1). We, however, were able to discriminate alterations of sizes related with the purification procedure. Unpurified materials were characterized with slightly bigger diameter in comparison with materials that were subjected to purification. We hypothesize that inertial shear forces present during purification were responsible for breaking some of the flakes to smaller ones.

Our SEM findings were further justified with an atomic force microscopy study. AFM has proven to be one of the most prominent methods used to identify flakes of graphene and its derivatives. One can find literature reports estimating the thickness of single-layer graphene in the range of 0.4–1.7 nm [35,36,37]. Moreover, the AFM investigation presented in this study also stays in good correlation with data provided by the literature, indicating an almost complete exfoliation of graphite oxide into individual graphene oxide flakes. The examination of at least 50 flakes per sample revealed that c.a. 91% of the sheets were actually single-layer GO (with average thickness in the range of 0.9–1.7 nm), whereas the remaining 9% corresponded to GO stacks, containing two or more GO layers (GO agglomerates were not included in the analysis). Therefore, the as developed protocol of preparation allowed us to obtain almost full exfoliation of GO into individual flakes. Fabrication of single flakes is crucial as most of the GO’s exceptional properties are combined with their thickness. We want to emphasize that the statistical information about the exfoliation of the GO flakes was recorded exclusively for purified samples (fresh and aged).

**Figure 3:**
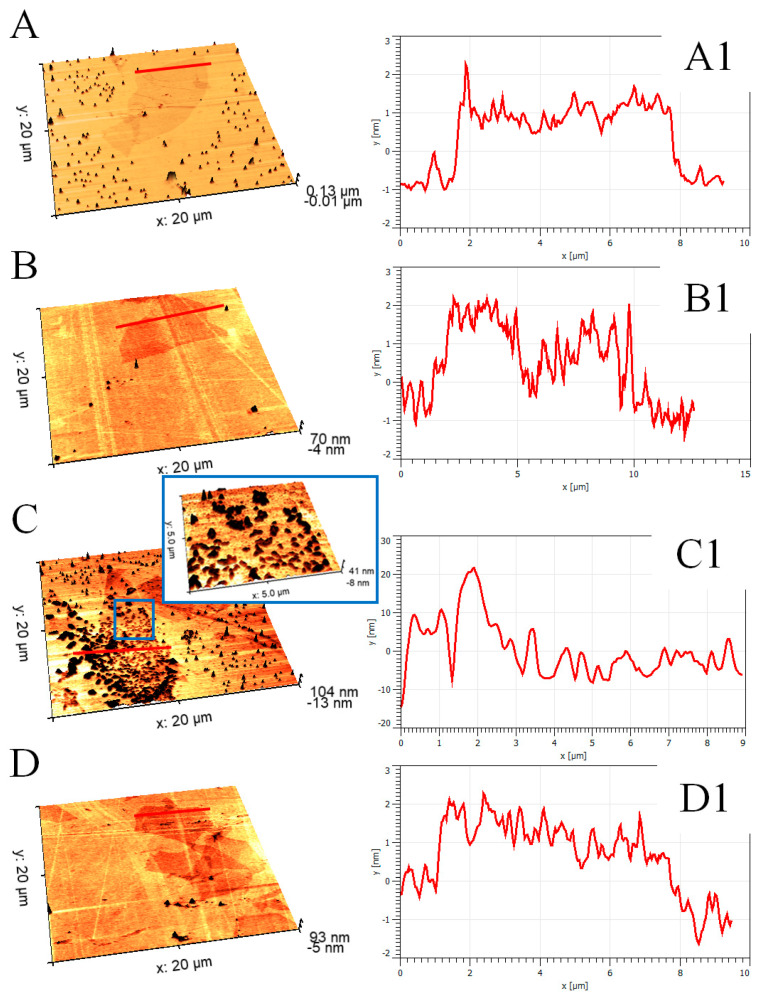
AFM topographical images of graphene oxide flakes: (**A**) as-obtained, (**B**) purified, (**C**) aged_as-obtained, (**D**) aged_purified, accompanied with their topographical profiles on images (**A1**,**B1**,**C1**,**D1**). The scanning area is 20 × 20 µm. Red markers correspond to a place from which topographical profiles were taken.

High-resolution AFM maps revealed many impurities (dark spots on AFM topography images, Figure 3) in case of as-obtained and aged_as-obtained samples. In the most prominent case (Figure 2C), almost the whole GO flake was covered with impurities, which altered the flake’s profile to a large degree (from 1.2 nm to 30 nm in height). Interestingly, in the case of purified samples, only slight contaminations were registered, and even after 14 months of storage, the flakes were relatively free of impurities. Interestingly, all impurities were located in the solution (outside the flakes) or on the surface of GO flakes, rather than on their edges, which may indicate different properties of flakes’ edges compared to GO’s surface. Such a conclusion cannot be surprising as a congruent phenomenon was widely discussed by Lerf et al. [38], who stated that carbonyl and carboxylic groups are attached to the flakes’ edges in contrary to the hydroxyl and epoxy groups attached to the surface of the flakes. Further studies were performed by Yuan et al. [39] and have proved that this dissimilarity impacts the electrochemical behavior of graphene flakes. However, it is worth underlining that in the case of as-obtained GO flakes, impurities were present rather in solution than on the surface of flakes. On the contrary, after 14 months of aging, most of the investigated flakes were covered with impurities.

**Figure 4:**
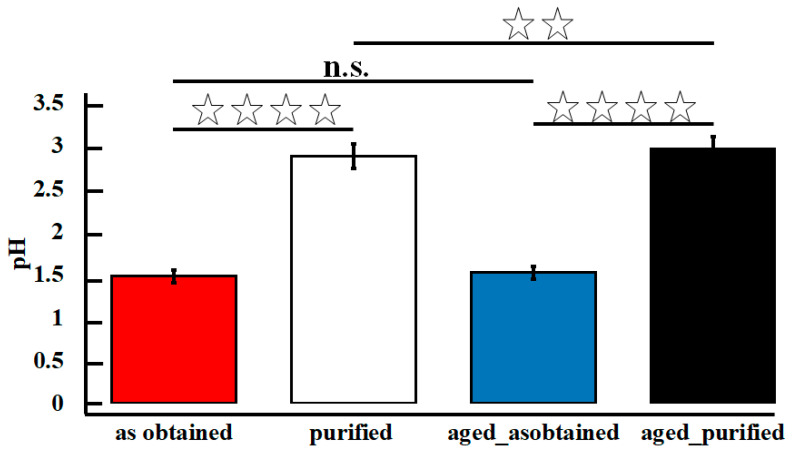
Results pH measurements for the evaluated samples. Differences were assigned as significant for: * *p* < 0.05, ** *p* < 0.01, *** *p* < 0.001, **** *p* < 0.0001.

To check the time-dependent alterations of GO performance, we have conducted pH examination (Figure 4). One should know that a pH plays a pivotal role in GO hydrophobicity, which in turns controls agglomeration and antibacterial properties of GO flakes. Barbolina et al. showed that antibacterial properties of GO are most likely explained by their acidic pH [40]. At the same time, low pH makes GO flakes less hydrophilic, and their agglomeration is widely observed [41]. Our results showed that as-obtained and aged_as-obtained samples were characterized with the lowest pH with the value of 1.48, which increased after the purification. Such observation was expected as unpurified material contains acidic residues coming from sulfuric acid. At the same time, it should be noted that the acidic nature of GO aqueous solution is not exclusively driven by the post-production impurities. One should remember, that GO flakes are characterized with the presence of acidic functional groups on their surface, like carboxylic acid group (COOH). The overriding conclusion from our data is that all examined GO-based water solutions were acidic, and neither the purification step, nor the storage influenced their acidity. This might be of potential interest in case of application of GO flakes as antibacterial agents. We want to emphasize, that in the frame of presented study, the pH should be mostly considered as an indicators of successful purification of the material.

**Figure 5:**
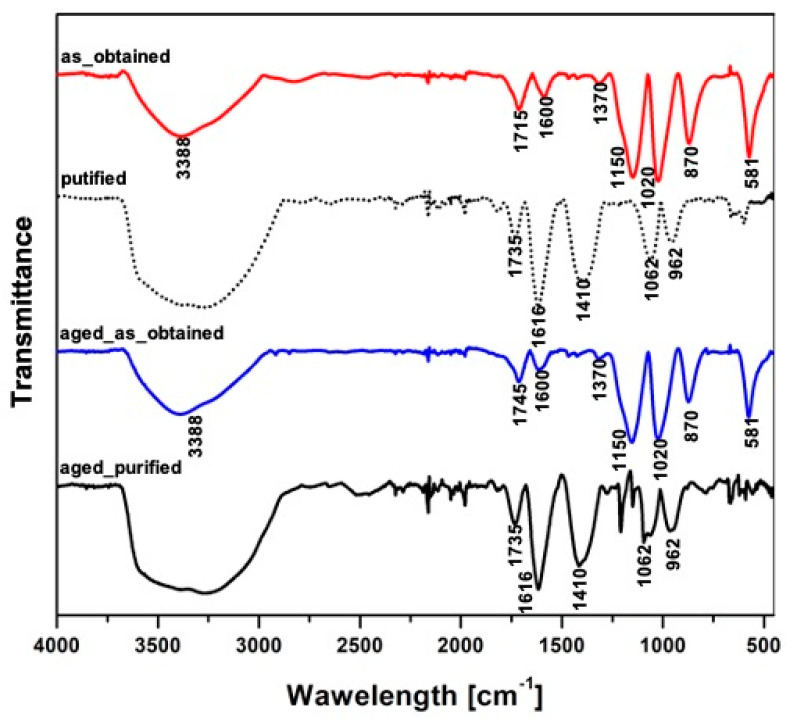
FTIR spectra of the as-obtained, aged_as-obtained, purified and aged_purified samples.

FTIR investigation allowed detecting all characteristic groups assigned to graphene oxide, with shifts connected to the purifications process (Figure 5). Peaks observed for all the registered spectra between 1745 cm^−1^ and 1715 cm^−1^ are attributed to the stretching vibrations of carbonyl groups, which denotes carboxyl, lactone, and quinone formed during the oxidation of the graphite. The transmission bands present near 1616 cm^−1^ can be assigned to water that has been physisorbed by hydrogen bonds [42]. A peak connected to C-O bonding can be observed at 1150 cm^−1^, and C-OH or C-O-C stretching vibration-related peak is present at 1410 cm^−1^ or 1370 cm^−1^ [43]. While further comparing the spectra of non-purified and purified graphene in the case of as_obtained and aged_as-obtained samples, sharp peaks at 860 cm^−1^ and 580 cm^−1^ were observed, which might be related residuals of oxides of manganese [44]. Manganese dioxides (detected with FTIR—Figure 5) are considered to have good electrical conductivity [45], and it is reasonable to assume that enhanced ionic conductivity is driven by these post preparation impurities.

**Figure 6:**
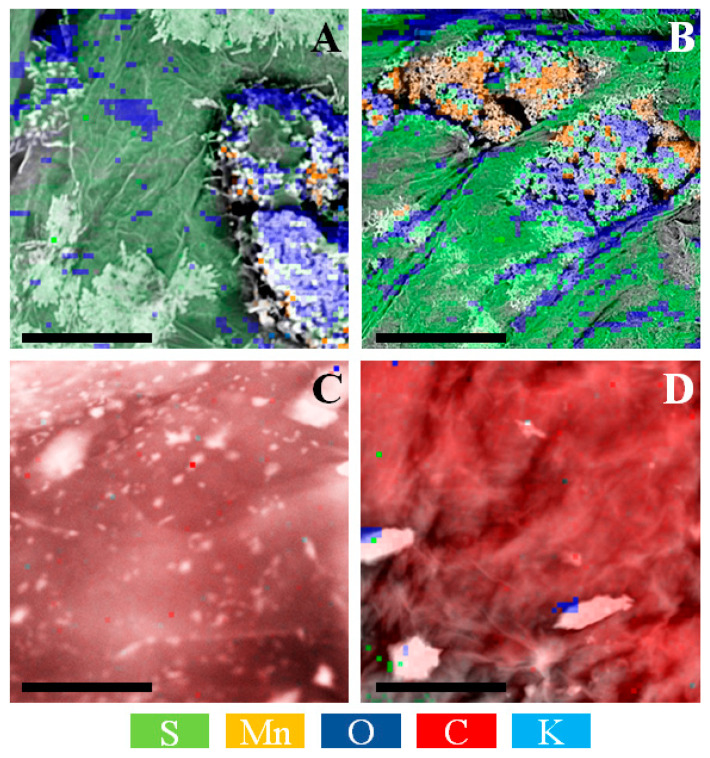
EDS maps of: (**A**) as-obtained, (**B**) aged as-obtained, (**C**) purified, (**D**) aged purified samples. The scalebar is 200 µm.

Moreover, the presence of manganese and potassium was registered on EDS elementals’ maps (Figure 6), which confirms the extant presence of reagents used for the fabrication of graphene oxide using the modified Hummers method. Purified samples were composed of only carbon, oxygen, and sulfur traces. This hypothesis is supported by the literature reports, proving that graphene-derivatives doped with potassium exhibit enhanced electrical performance [46]. In order to quantify the amount of carbon, oxide, and sulfur, elemental combustion analysis was implemented (Table 1).

It is worth underlining that elemental combustion analysis allows to precisely determine even a meager number of elements in the tested material. However, the detection is limited and strictly defined by the installed detector; thus, we were only able to quantify the percentage amount of carbon, oxygen, and sulfur (the amount of manganese and potassium detected by the EDS was not established). The data presented in Table 1 showed that the purification procedure efficiently decreased sulfur concentration. At the same time, it can be hypothesized that the impurities in the synthesized (unpurified) material were present in the oxygenated condition as the material deprived of purification was characterized by an elevated amount of oxygen compared with its purified form. We can conclude that 14 months of aging did not influence the electrical performance of tested samples.

**Figure 7:**
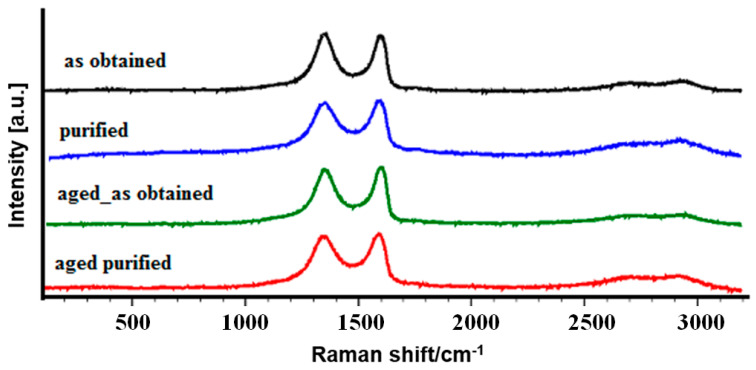
Raman spectra of GO samples tested in this study.

Raman analysis confirmed the presence of intensive D (1348–1353 cm^−1^) and G (~1590–1600 cm^−1^) bands (Figure 7, Table 2) typical for GO at all samples. The G band is associated with in-plane vibrations of sp2 bonded carbon atoms, whereas the D band is attributed to the presence of defects and disorder such as in-plane hetero-atoms, grain boundaries, and aliphatic chains. Additionally, small peaks approximately at ~2713 cm^−1^ and ~2930 cm^−1^ can be attributed to the overtone of D and D + G bands [27]. According to the results presented in Table 2, both the G and the D bands shifts towards lower wavenumbers and widen after purification process what indicates on increasing amount of the disordered phase after purification process [47]. The reduction of ID/IG ratio observed for purified samples could suggest that most of the oxygen-containing groups have been removed, resulting in the recovery of sp2 carbon–carbon bonds [27]. Only slight changes of peaks position, FWHM increase, and the ratio of ID/IG (Table 2) were observed after aging time, which indicates that the structure of GO was not influenced by this process.

**Figure 8:**
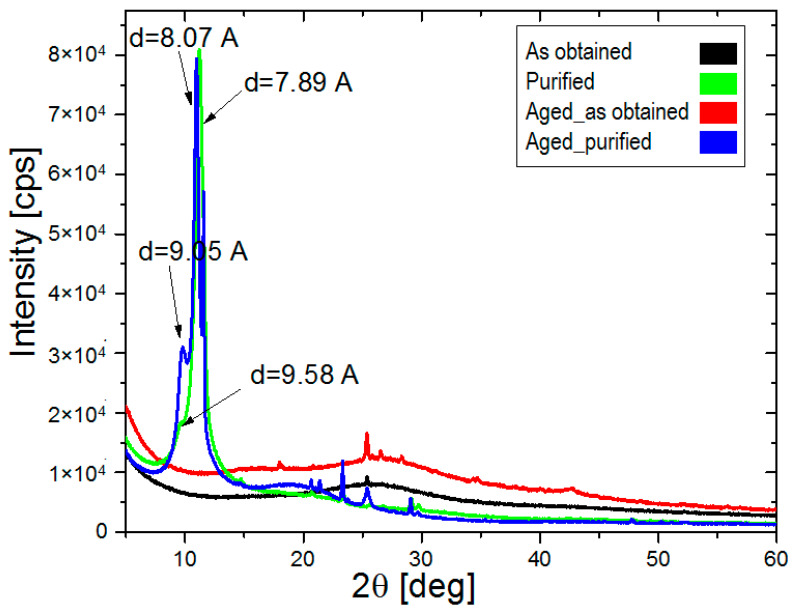
XRD diffractograms of synthesized materials.

It is interesting to note that purified samples were characterized with bimodal structure—presumably related with intercalated graphene flakes. The arrows indicated inter-layer distances (Figure 8). At the same time, we want to report that samples deprived of purification were characterized with a typical amorphous structure. Detailed analysis of the obtained diffractograms allowed us to spot additional narrow reflexes, which originated from post-production impurities. The registered XRD-data enabled us to state that the purification protocol was successful.

The overriding conclusion from presented work is that long-term storage in ambient conditions in the form of purified suspension did not cause reduction of pristine GO flakes, the properties of which were well preserved. At the same time, it is worth noting that our findings polarize with seminal publications written by Du et al. and Dimiev et al. [48,49]. Dimiev et al. proposed a novel description model of GO, in which (against all formerly models), GO was reviewed as a material characterized with unstable functional groups, constantly developing under water conditions. In addition, they claimed that after 2 months of GO flakes exposition on water, the flakes started to degrade. We, however, were not able to spot any differences in flakes morphology, nor their lateral size after 14 months of exposition to water. In addition, Du et al. argued that during long-term storage at room temperature GO flakes tend to be chemically unstable. They mentioned that this chemical is connected with the removal of oxygenated groups from GO surface. In fact, our results of combustion elemental analysis allowed us to observe a similar trend (Table 1). To overcome this problem, they proposed a new solvent-propylene carbonate and proved GO solution stability over a period of 28 days, which is a much shorter time than in the case of our study. In addition, Yeh et al. [50] wrote a paper mentioning the stability of GO membranes in water, in which it was proven that GO based filters are characterized with different mechanical properties and stability after exposition to aqueous environment—they concluded that such a material is unstable. They proposed crosslinking of GO-based membranes with multivalent cationic metal contaminant as an attractive method to enhance mechanical performance and stability of the tested membranes. Having in mind the material tested in our study, it is worth emphasizing that we tested exclusively a few-layered GO flakes, not a thin film, or a bulk material, which in fact could be dissolved in water. The reason for the difference between our results and results depicted in some publications may be attributed with different preparation and purification protocol—in fact various laboratories around the world use alternative fabrication methods (also different graphite precursors), which may result in different properties of a final product. The question remains—does a slight change of oxygen content makes a material unstable? We did not observe GO flakes’ degradation, or flakes dissolving in water. However, further studies are needed to explain this contradiction.

## 4. Conclusions

Graphene oxide flakes were synthesized and then aged in ambient conditions for over 14 months in order to check whether storage time impacts their purity. AFM topography maps revealed almost complete exfoliation of graphite oxide into a few layers of graphene oxide flakes, with thickness in the range of 0.9–1.7 nm. The morphology of samples deprived of purification was affected by the presence of impurities, which led to an increase in the thickness of individual flakes, even up to 30 nm. Storage time was followed by the increased concentration of impurities present on the surface of the flakes. The overall conclusion that might be driven from the current research is that long-term storage in ambient conditions in the form of purified suspension did not cause any alterations or reduction of pristine GO flakes, the properties of which were well preserved. GO material synthesized and tested in this study was stable and the purification protocol was successful.

## Figures and Tables

**Table 1:** Percentage of elements in the material.

Samples	Carbon [%]	Oxygen [%]	Sulphur [%]
as-obtained	15.3 ± 0.2	73.7 ± 0.3	7.6 ± 0.2
purified	41.7 ± 0.2	45.7 ± 0.2	2.4 ± 0.2
aged_as-obtained	14.7 ± 0.1	69.7 ± 0.3	8.9 ± 0.2
aged_purified	40.6 ± 0.2	44.2 ± 0.2	3.2 ± 0.1

**Table 2:** Raman shift positions, full width in the high maximum (FWHM) and intensity ratio (ID/IG) of GO films.

Samples	Peaks Position, Raman Shift/cm^−1^		FWHM, cm^−1^		ID/IG
	D Band	G Band	D Band	G Band	
as-obtained	1351	1598	86	64	1.027
purified	1350	1591	99	74	0.869
aged_as-obtained	1354	1600	91	66	0.946
aged_purified	1348	1590	101	73	0.904

## Data Availability

Data are contained within the article.

## Supplementary Materials

The following are available online at https://www.mdpi.com/article/10.3390/ma14154108/s1, Figure S1: A histogram depicting distribution of a diameter (lateral size) of synthesized materials.
