# Supplementary material for: Morphology and Chemical Purity of Water Suspension of Graphene Oxide FLAKES Aged for 14 Months in Ambient Conditions. A Preliminary Study
Source: Materials (Basel). 2021 Jul 23;14(15):4108. doi: 10.3390/ma14154108 (PMC8347880; doi:10.3390/ma14154108)
Supplement: Supplementary file 1 [file materials-14-04108-s001.zip › materials-1264297-supplementary.pdf]

materials

MDPI

Morphology and Chemical Purity of Water Suspension of Graphene Oxide FLAKES Aged for 14 Months in Ambient Conditions. A Preliminary Study

Adrian Chlanda [1,*,†], Krystian Kowiorski [2,†], Marcin Małek [3], Ewa Kijeńska-Gawrońska [4,5], Monika Bil [4,5], Małgorzata Djas [1,6], Tomasz Strachowski [1], Wojciech Swieszkowski [5] and Ludwika Lipińska [1]

1 Łukasiewicz Research Network-Institute of Microelectronics and Photonics, Department of Graphene and Composites, Aleja Lotników 32/46, 02-668 Warsaw, Poland; Malgorzata.Djas@imif.lukasiewicz.gov.pl (M.D.); tomasz.strachowski@imif.lukasiewicz.gov.pl (T.S.); Ludwika.Lipinska@imif.lukasiewicz.gov.pl (L.L.)
2 Łukasiewicz Research Network-Institute of Microelectronics and Photonics, Department of Functional Materials, Aleja Lotników 32/46, 02-668 Warsaw, Poland; krystian.kowiorski@imif.lukasiewicz.gov.pl
3 Faculty of Civil Engineering and Geodesy, Military University of Technology, Gen. Sylwestra Kaliskiego 2, Warsaw, Poland; marcin.malek@wat.edu.pl
4 Centre for Advanced Materials and Technologies CEZAMAT, Warsaw University of Technology, Poleczki 19, 02-822 Warsaw, Poland; ewa.kijenska@pw.edu.pl (E.K.-G.); monika.bil@pw.edu.pl (M.B.)
5 Faculty of Materials Science and Engineering, Warsaw University of Technology, Biomaterials Group, Wołoska 141, 02-507 Warsaw, Poland; wojciech.swieszkowski@pw.edu.pl
6 Faculty of Chemical and Process Engineering, Warsaw University of Technology, Waryńskiego 1, 00-645 Warsaw, Poland
* Correspondence: adrian.chlanda@gmail.com or adrian.chlanda@imif.lukasiewicz.gov.pl
† Contributed equally.

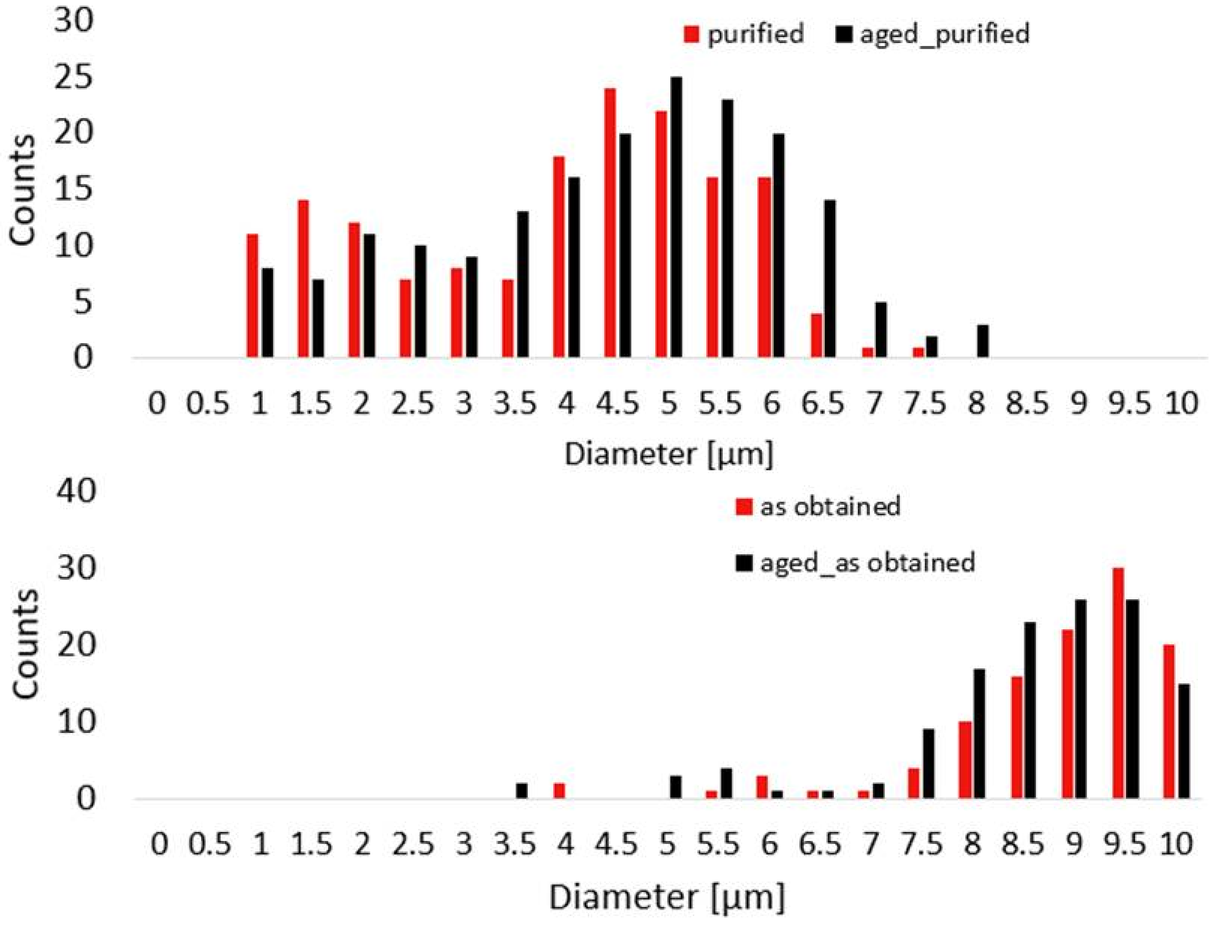

Figure S1. A histogram depicting distribution of a diameter (lateral size) of synthesized materials.
